# Natural Sirtuin1 Activators and Atherosclerosis: an Overview

**DOI:** 10.1007/s11883-023-01165-4

**Published:** 2023-12-01

**Authors:** Karolina Łanoszka, Nimasha Vlčková

**Affiliations:** Department of Human Nutrition and Dietetics, Faculty of Food Technology, University of Agriculture in Krakow, 122 Balicka Street, 30-149 Krakow, Poland

**Keywords:** SIRT1, Natural compounds, SIRT1 activators, Atherosclerosis

## Abstract

**Purpose of Review:**

The purpose of this review is to summarize the most recent findings investigating the impact of several natural sirtuin (SIRT) activators, particularly SIRT1, on atherosclerosis.

**Recent Findings:**

Sirtuins that belong to a family of class III histone deacetylases are believed to be novel therapeutic targets to treat age-related and chronic diseases. SIRT expression is regulated by small molecules called SIRT-activating compounds that can be found in natural food products. SIRT1 may exert protective effects in atherosclerosis, which is said to be a major cause of cardiovascular diseases. Most of the evidence supporting the beneficial effects of these natural compounds comes from in vitro or animal-based studies, while there have been particularly few or inconsistent human-based studies evaluating their long-term impact in recent years.

**Summary:**

SIRT1 activation has been demonstrated to mitigate or prevent atherosclerosis through various mechanisms. However, further research is required to determine the optimal SIRT activator dosage and to establish a stronger correlation between health effects and the administration of bioactive compounds. Additionally, conducting more human clinical trials is necessary to ensure the safety of these compounds for preventing atherosclerosis development.

## Introduction

Noncommunicable diseases (NCDs) kill 41 million people each year, which is equivalent to 71% of all global deaths, as stated by the World Health Organization. Annually, more than 15 million people die from NCDs between the ages of 30 and 69; 85% of these “premature” deaths occur in low- and middle-income countries. Cardiovascular diseases (CVDs) account for the majority of NCD-related deaths, with 17.9 million people succumbing to them each year, followed by cancers (9.3 million), respiratory diseases (4.1 million), and diabetes (1.5 million). Atherosclerosis is the primary underlying cause of most cardiovascular diseases [1].

Atherosclerosis is the most common form of CVD, where the disease’s main components are lipid accumulation and inflammation of the large arteries. These factors can eventually lead to clinical complications, such as myocardial infarctions (MIs) and strokes [2]. The exact causes and risk factors of atherosclerosis are not fully understood. However, certain conditions, traits, or habits may increase the likelihood of developing atherosclerosis. High levels of total cholesterol (TC) and low-density lipoprotein (LDL) levels, along with low levels of high-density lipoprotein (HDL) in the blood, hypertension, exposure to tobacco smoke, diabetes mellitus, obesity, and a sedentary lifestyle are all risk factors. Therefore, atherosclerosis can be delayed or prevented by controlling these risk factors.

Members of the sirtuin (SIRT) family of proteins are class III histone deacetylases that are homologous to the yeast silent information regulator 2 (Sir2). Sirtuins mediate the deacetylation of histones and non-histone proteins in an NAD^+^-dependent manner. SIRT1 was the first SIRT to be discovered in mammals and is the most extensively studied SIRT protein, playing a role in promoting longevity [3].

Over the past few years, a healthy and balanced diet has been encouraged to prevent diseases in humans [4, 5], atherosclerosis, and its development in particular [6–8]. Food bioactive compounds are extra nutritional constituents that typically occur in small quantities in foods [9]. Bioactive compounds in the diet can act as antioxidants and anti-inflammatory agents, reducing the negative effects of oxidative stress and the incidence of chronic diseases, such as obesity, diabetes, and cardiovascular disorders [9–11]. The beneficial effects of consuming foods rich in polyphenols have been widely discussed in relation to cardiovascular diseases, including atherosclerosis, high blood pressure, thrombotic diseases, stroke, or hyperlipidemia [12].

Many in vitro [13–15], animal [16, 17], and human studies [18, 19] have shown that SIRT1 has anti-atherogenic properties. Several plant-based bioactive compounds have demonstrated their ability to modulate SIRT1. These compounds are found in many plants and foods, such as fruits, vegetables, tea, cereals, and wine, and long-term intake is associated with health benefits [20]. Interesting in vivo (Table 1) [21–31], in vitro (Table 2) [22–24, 30, 32–35], and human (Table 3) [36–39] clinical trial studies have shown their effects on atherosclerosis development and risk factors associated with atherosclerosis.

**Table 1:** Natural SIRT1 activators and their effect on atherosclerosis development in vivo

	Molecular pathway	Model	Dose and duration	Control	Cardiovascular effect	Reference
Quercetin	AMPK/SIRT1/NF-κB signaling pathway	Diabetes-induced Wistar rats fed a high-fat diet	30 mg/kg/day, orally for 2 weeks	Male Wistar rats	↓ atherosclerosis lesions ↓ MDA levels ↓ NF-κB and IL-1β levels ↑ IL-10 levels	[21]
	↑ SIRT1 in aorta	Male ApoE^−/−^ mice fed a high-fat diet	20 mg/kg/day, intragastrically for 8 weeks	Wild-type C57BL/6 J	↓ lipid deposition in arterial lumina ↓ serum ICAM-1, IL-6, and VCAM-1 in aorta	[22]
Berberine	↑ Kruppel-like factor (KL) expression through KL/SIRT1 signaling pathway	Male Sprague Dawley rats	Aged rats without berberine Aged rats with low-dose berberine (100 mg/kg) Aged rats with high-dose berberine (200 mg/kg) Feeding with diet containing berberine for 2 months	Young male Sprague Dawley rats for each group of aged rats	↓ aging markers ↑ expression of KL ↓ hypertrophy-associated genes ANP and MHC in the aging heart Protected the aging heart	[23]
	SIRT1-mediated p66Shc suppression	Sprague Dawley rats	DOX with berberine 10 mg/kg and 20 mg/kg orally administered for 10 days	Control; 0.9% saline DOX treatment; 20 mg/kg; intraperitoneal injections of DOX (20 mg/kg/day diluted with 0.9% saline) every other day for a total of 3 injections	↑ catalase ↑ superoxide dismutase ↑ glutathione peroxidase activities ↓ MDA Improved the electrocardiogram and histopathological changes in the myocardium	[24]
Fisetin	↓ PCSK9 ↓ LOX-1 ↓ p21 ↓ p53 ↓ p16	ApoE^−/−^ mice fed a high-fat diet	ApoE^−/−^ mice fed a high-fat diet and aqueous solutions of fisetin 12.5 mg/kg daily for 12 weeks via oral gavage	C57BL/6 wild-type mice fed a normal mouse diet	↓ atherosclerotic lesions ↓ lipid accumulation	[25]
	AMPK/SIRT1 pathway	Male ICR mice	Pb group (lead acetate (200 mg/L) dissolved in the drinking water), Pb + fisetin 25 mg/kg bw and Pb + fisetin 50 mg/kg bw dissolved in 0.1% dimethyl sulfoxide, intragastrically once daily for 4 weeks	Saline 0.9% NaCl tragastrically daily	↓ inflammation	[26]
Curcumin	↑ SIRT1 ↑ HO-1	C57BL/6 J mice	8-week-old C57BL/6 J mice were fed a high-fat diet with 0.1% curcumin for 74 weeks	8-week-old C57BL/6 J mice were fed a high-fat diet for 74 weeks	↓ blood glucose levels ↓ TC Enhancement of HO-1 in aorta ↓ production of superoxide in the thoracic aorta ↓ regional aortic oxidative stress and systemic oxidative stress	[27]
	SIRT1-Foxo1 and PI3K-Akt pathways	Male Sprague Dawley rats with streptozotocin-induced diabetes fed a high‐glucose and high‐fat diet	4-week-old diabetes-induced male Sprague Dawley rats fed a high‐glucose and high‐fat diet, 100 mg/kg/day orally administered	Male Sprague Dawley rats fed a high‐glucose and high‐fat diet	↓ blood glucose levels Improved cardiac dysfunction Delayed the onset of cardiovascular complications ↓ oxidative stress Inhibition of cardiomyocytes apoptosis	[28]
	↑ SIRT1	Male Wistar rats	1 mg kg/day, orally for 15 days	Male Wistar rats treated with water	↓ aortic vasodilation ↓ blood pressure	[34]
	H_2_O_2_ inhibition eNOS restoration	Wistar rats fed ox-LDL or phosphatidylcholine hydroperoxide (PCOOH)	10 mg/kg/day, intragastrically for 2 weeks	Wistar rats fed ox-LDL or PCOOH	↓ ox-LDL/PCOOH induced vasoconstriction ↓ urinary 8-isoprostane levels ↓ ICAM-1 expression ↓ 4-HNE accumulation Restored nitrite/nitrate amounts	[30]
Resveratrol	Inhibit the proliferation and activation of CD4 + T	ApoE^−/−^ mice fed a high-fat diet with lipopolysaccharide and simvastatin	5 mg/kg (bw)/day of RSV dispersed in 5% sodium carboxymethyl cellulose solution administered intragastrically daily for 20 weeks	ApoE^−/−^ mice fed a normal mouse diet with 5% sodium carboxymethyl cellulose solution	↓ TC ↓ TG ↓ LDL-C ↓ non-HDL-C ↑ HDL-C ↓ infiltrated lesion of the aorta ↓ plaque area ↓ CD4 + T cell in peripheral blood mononuclear cells ↓ CD25 and CD44 expression	[31]

**Table 2:** Natural SIRT1 activators and their effect on atherosclerosis development in vitro

	Molecular pathway	Model	Dose and duration	Control	Cardiovascular effect	Reference
Quercetin	Nitrogen metabolism, ECM-receptor interaction, complement and coagulation cascades, p53, and mTOR signaling pathway	HAECs induced by ox-LDL	HAECs cultured for 48 h in the presence of 50 µg/ml ox-LDL or 50 µg/ml ox-LDL with quercetin; concentrations of 3 µmol/L, 1 µmol/L, or 0.3 µmol/L	Untreated HAECs	↓ expression of senescence-associated β-galactosidase ↓ improved cell morphology cellular apoptosis ↑ mitochondrial membrane potential ↓ ROS generation	[22]
Berberine	↑ Kruppel-like factor (KL) expression through KL/SIRT1 signaling pathway	Senescent H9c2 cells (a ventricular cardiomyocyte cell line) induced by doxorubicin (DOX)	H9c2 cardiac cells exposed to DOX for 24 h with berberine	H9c2 cardiac cells exposed to DOX for 24 h without berberine	Protected H9c2 cells against DOX-induced senescence ↓ levels of ROS species Inhibited apoptosis Alleviated mitochondrial dysfunction	[23]
	SIRT1-mediated p66Shc suppression	Rat cardiac H9c2 cell line	Pretreated H9c2 cardiac cells with 0.1 µM, 1 µM, or 10 µM berberine for 24 h, later exposed to 1 µM DOX for 24 h	H9c2 cardiac cells exposed to DOX for 24 h without berberine pretreatment	Improved DOX-induced oxidative insult and mitochondrial damage by adjusting the levels of intracellular ROS, mitochondrial membrane potential (ΔΨm), and mitochondrial Ca^2+^ ([Ca^2+^]m) in H9c2 cells	[24]
Fisetin	↓ MCP-1 ↓ IL-1β ↓ iNOS ↓ uPA ↓ uPAR	Macrophages obtained from BALB/c mice stimulated with lipopolysaccharide	Fisetin 10 µM, 30 µM, and 100 µM for 3 h	A vehicle control	↓ inflammatory responses ↓ formation of foam cells	[32]
	↑ SIRT1	HUVECs induced by doxorubicin	2 µg/ml for 3 h	HUVECs induced by doxorubicin without curcumin	↓ IL-1β ↓ IL-6	[33]
	↑ SIRT1	Human THP-1 cells induced by lipopolysaccharides	2 µg/ml for 3 h	Human THP-1 cells induced by lipopolysaccharides without curcumin	↓ TNF-α	[33]
Catechins	↑ eNOS ↑ SIRT1	Bovine coronary artery endothelial cells (BCAEC)	1 µM for 48 h	BCAEC with water	↓ cell senescence (β-galactosidase) ↑ NO production	[34]
	H_2_O_2_ inhibition eNOS restoration	HUVECs induced by phosphatidylcholine hydroperoxide (PCOOH)	100 µg/ml catechins before PCOOH treatment	HUVECs induced by PCOOH without catechins	↓ PCOOH-elevated H_2_O_2_ ↓ apoptosis of endothelial cells ↓ ROS production	[30]
Resveratrol	↓ MMP-9 ↑ TIMP-1	Human aorta-vascular smooth muscle cells (HA-VSMCs) treated with H_2_O_2_	80 µmol/L, 100 µmol/L, and 120 µmol/L RSV for 24 h and 48 h	HA-VSMCs not treated with H_2_O_2_	↓ MMP-9 expression ↑ TIMP-1 production	[35]

**Table 3:** Natural SIRT1 activators and their effect on atherosclerosis development in human

	Molecular pathway	Model	Dose and duration	Control	Cardiovascular effect	Reference
Resveratrol	↑ PPARγ ↑ SIRT1	56 patients with type 2 diabetes mellitus (T2DM) and coronary heart disease	500 mg resveratrol (RSV) per day (*n* = 28) for 4 weeks	Placebo group (*n* = 28) for 4 weeks	↓ fasting glucose ↓ insulin ↓ insulin resistance ↑ insulin sensitivity ↓ TC-to-HDL-C ratio ↓ MDA ↑ HDL-C ↑ total antioxidant capacity	[36]
	↑ circulating SIRT1 in serum	48 healthy adults (24 women and 24 men), aged 55 to 65 years	RSV 500 mg/day for 30 days	Energy restriction 1000 kcal/day for 30 days	↑ TC ↓ apoB ↓ noradrenaline	[37]
	↑ SIRT1	97 older adults with type 2 diabetes	RSV 1000 mg/day for 6 months	Placebo group for 6 months	1000 mg/day group results: ↑ total antioxidant capacity ↑ antioxidant gap ↓ TG-C No change in CRP ↓ MDA ↓ 8-isoprostane ↑ SOD	[38]
Catechin	↑ SIRT1 in serum	30 overweight young women (aged 20–30 years old and body mass index > 25 kg/m^2^)	High-intensity interval training (HIIT) + green tea tablets of 1500 mg Green tea tablets for 10 weeks, 7 days/week, 3 times/day, 2 h after main meals	HIIT + placebo 1500 mg starch powder tablets for 10 weeks, 7 days/week, 3 times/day, 2 h after main meals Green tea 3 × 500 mg for 10 weeks, 7 days/week, 3 times/day, 2 h after main meals	↑ CAT compared to green tea and HIIT + placebo ↑ PGC-1α compared to green tea and HIIT + placebo Improved antioxidant system, body composition, and VO_2_ max compared to green tea and HIIT + placebo	[39]

In the present review, we discuss novel insights into the effects of natural molecules considered as SIRT1-activating compounds and their impact on atherosclerosis in the last 5 years.

## SIRT1 Activators and Atherosclerosis

### Resveratrol

Resveratrol (3,5,4′-trihydroxystilbene) is a polyphenolic chemical compound found in food products such as grapes, peanuts, and berries. It is biosynthesized in response to pathogens. Red wine is also a resveratrol-rich product, as the winemaking process involves crushing and mashing grapes, which leads to its release from the fruit [40]. The concentration of resveratrol in red wines ranges from 0.1 to 14.3 mg/L. Its concentration in red grape juice, fresh grape skin, and grapes (dry sample) is 0.5 mg/100 ml, 5–10 mg/100 g, and 0.64 mg/100 g, respectively [41]. Resveratrol exists in two isomeric structures: the *cis* and *trans* isoforms. The *trans* isoform can be converted to the *cis* isoform through heating [40]. Oral intake of high doses of resveratrol (5 g) from resveratrol-containing foods or supplements has been demonstrated to be safe, with no serious adverse effects reported. The concentration of unmetabolized resveratrol found in plasma after oral administration of various resveratrol-rich sources in various studies did not exceed 5 µM and ranged from plasma resveratrol levels of 1.8 to 4.2 µM [42]. Numerous studies have demonstrated its anti-aging and anti-inflammatory effects both in vivo (using yeasts, insects, mice, and other organisms) and in vitro (utilizing both animal and human cell lines)[43, 44].

Resveratrol treatment has been shown to reduce atherosclerosis in numerous in vivo studies using various animal models. Dietary enrichment with resveratrol resulted in a reduction in the size of atherosclerotic lesions [31, 45, 46]. A study demonstrated its protective effect on vascular structure by showing that resveratrol prevented TNF-α-induced damage to elastin fibers in aortic cross sections of C57BL/6 mice [42]. Many experimental studies using atherosclerosis mouse models have shown that resveratrol lowers TC, TG, LDL-C, and very-low-density lipoprotein (VLDL)-C levels while increasing HDL-C [31, 45]. In contrast, a randomized clinical trial involving human subjects with dyslipidemia provided a counterexample to the aforementioned reports. During an 8-week supplementation period with resveratrol (at doses of 100 mg/day, 300 mg/day, or 600 mg/day), there were no significant changes observed in the lipid profile when compared to the placebo, regardless of the dosage used. The researchers noted no significant differences in triglycerides, TC, HDL and LDL cholesterol, apoA1, apoB, or apoA1/apoB between baseline and follow-up in the four groups [47]. The anti-atherogenic effect of resveratrol was demonstrated in ApoE^−/−^ mice, where a reduction in macrophage infiltration was observed in animals treated with resveratrol [31]. Resveratrol was found to reduce the expression and serum levels of various chemokines and adhesion molecules, such as CCL2, CXCL1/KC, MCP-1, ICAM-1, and VCAM-1, in both mice and human endothelial cells, as well as in THP-1 human monocytes. This effect has been demonstrated to influence the recruitment and adhesion of circulating blood monocytes [42, 46]. Atherosclerosis is regarded as an inflammatory disease, making compounds with anti-inflammatory properties, such as resveratrol, of paramount importance in the prevention of vascular atherosclerosis and the subsequent cardiovascular diseases that may arise [48]. The in vivo part of the experimental study, a type of study, first demonstrated that resveratrol attenuated vascular endothelial inflammation by reducing VCAM-1 and F4/80 expression in aortic cross sections of C57BL/6 mice after TNF-α stimulation. This effect was achieved through the inhibition of NF-κB factor activation [42]. A study using ApoE^−/−^ mice and umbilical vein endothelial cells (UVECs) isolated from ApoE^−/−^ mice demonstrated several positive effects of resveratrol. These effects included reduced atherosclerotic plaques; lower levels of TC, TG, LDL-C, and HDL-C; as well as decreased levels of TNF-α, C-reactive protein, matrix metallopeptidase 9 (MMP-9), and CD40L expression in arterial lesion tissue. The study suggested that resveratrol’s anti-atherosclerotic properties were attributed to the modulation of the PI3K/AKT/mTOR pathway [45]. CD4 + T cells, which are present in atherosclerotic lesions, play crucial roles in all stages of atherogenesis and have a significant impact on the regulation of the inflammatory process. A study demonstrated that the administration of resveratrol not only reduced atherosclerosis in vivo but also inhibited CD4 + T cell activation. Additionally, it reduced the expression of DNA-methyltransferase 1 (Dnmt1) and DNA-methyltransferase 3 beta (Dnmt3b) in CD4 + T cells [31]. In both animal- and human-based studies, resveratrol has demonstrated its ability to inhibit vascular smooth muscle cell (VSMC) proliferation induced by various mitogens. The specific molecular mechanisms involved depend on the type of mitogenic stimuli and may include the inhibition of the PI3K/Akt/mTOR pathway or cell cycle arrest [49].

In a randomized controlled trial involving adults with type 2 diabetes, researchers noted that resveratrol exhibited antioxidant properties and influenced markers of oxidative stress by activating SIRT1. They observed a significant reduction in markers of oxidative stress, and a more efficient antioxidant effect was evident in patients who received a resveratrol supplement at a dose of 1000 mg/day compared to those receiving 500 mg/day, which was associated with increased levels of SIRT1 [38]. In a recent study discussing the oxidative stress effects of resveratrol, it was demonstrated that the depletion of SIRT1 abolished the beneficial effects of resveratrol and pterostilbene (PTS), a natural methylated analog of resveratrol. This was observed in the context of protection against mitochondrial reactive oxygen species overproduction, mitochondrial dysfunction, and apoptosis in an H_2_O_2_-exposed intestinal porcine enterocyte cell line. These findings suggest that SIRT1 is essential for resveratrol and pterostilbene to protect against oxidative stress–induced intestinal injury [50]. In another recent study, it was demonstrated that resveratrol activated SIRT1 to enhance endothelial function in obese mice. This effect was achieved through the upregulation of peroxisome proliferator–activated receptor delta (PPARδ) expression in wild-type Ppard-wt mice on a high-fat diet [51]. PPARδ plays a significant role in lipid absorption, muscle endurance, insulin sensitivity, and the suppression of atherogenic inflammation [52]. Additionally, it was demonstrated that the SIRT1-mediated activation of PPARδ contributes to the beneficial effects of SIRT1 [51]. A study revealed that resveratrol suppresses insulin-induced VSMC proliferation and migration, potentially through the activation of SIRT1 and the downregulation of the PI3K/AKT pathway. This is supported by the fact that EX527, a specific inhibitor of SIRT1, nullified the role of resveratrol in inhibiting insulin-induced proliferation and migration while upregulating the phosphorylation of PI3K and Akt in VSMCs [53]. SIRT1 has been reported to prevent premature senescence of endothelial cells, thereby protecting them from dysfunction [54, 55]. In one study, the effect of resveratrol and its dimers, ε-viniferin and δ-viniferin, on NO production and wound repair in vascular endothelial cells was examined. All three compounds increased the wound repair of vascular endothelial cells (ECs) by promoting NO production and enhancing the expression of SIRT1 and heme oxygenase 1 (HO-1). These findings suggest a potential prevention of atherosclerosis development [56].

### Quercetin

Quercetin (3,3′,4′,5,7-pentahydroxyflavone) is the most common and widely distributed flavanol compound in our diet. It is commonly found in many fruits and vegetables, including apples, berries, red onions, grapes, cherries, broccoli, bell peppers, coriander, citrus fruits, and tea leaves (*Camellia sinensis*) [57, 58]. The estimated flavonoid intake ranges from 50 to 800 mg/day, with quercetin accounting for approximately 75% of that intake. This largely depends on the consumption of fruits and vegetables, as well as the intake of tea [59]. The bioavailability of quercetin is very low, primarily due to its extensive metabolism [60]. Another reason for its poor absorption is due to intestinal excretion [57]. Quercetin has antiradical activity due to the presence of reactive hydroxyl groups in its structure [61]. It reduces the formation of ROS by inhibiting NADPH oxidases and xanthine oxidases, decreases the activity of cyclooxygenases (COX) and lipoxygenases (LOX), and regulates the activity of intracellular signaling cascades involved in inflammatory reactions [62]. Quercetin metabolites are believed to be accumulated in tissues shortly after quercetin‐rich vegetables are consumed [63]. It was indicated that these metabolites, originating from enterocytes and the liver, serve as antioxidants by impeding oxidation of low‐density lipoprotein cholesterol [63].

Quercetin acts as an anti-inflammatory [60, 64–66] and anti-atherogenic [67–69] agent. Recent animal and in vitro studies have shown that quercetin reduces the size of atherosclerotic lesions [21, 22, 68, 70]. It has been suggested that excessive accumulation of oxidized LDL (ox-LDL) leads to an excessive inflammation in macrophages and a worsening condition of atherosclerosis by activating the nucleotide-binding oligomerization domain-like receptor protein 3 (NLRP3) inflammasome. It has also been noted that quercetin suppresses the galectin-3 NLR family pyrin domain containing 3 (Gal-3-NLRP3) proinflammatory signaling pathways in macrophages, subsequently alleviating atherosclerotic lesions [67]. Quercetin reduced the levels of IL-1β, TNF-α, IL-10, and IκBα gene expression, indicating a decrease in the transcriptional activity of NF-κB in individuals with coronary artery disease [62]. The reverse cholesterol transport (RCT) of macrophages in atherosclerotic plaques is a critical mechanism in the context of anti-atherosclerosis [71]. LXRα, SR-BI, and ABCA1 play a vital role in promoting macrophage RCT and in maintaining intracellular cholesterol homeostasis [58], and quercetin increased the expressions of PPARγ, LXRα, and ABCA1 genes in RAW264.7 macrophages exposed to ox-LDL [72]. Quercetin also attenuated the expression of PPARγ, LXRα, and ABCA1 in the aortas and livers of ApoE^−/−^ mice fed a high-fat diet [73]. Quercetin can alleviate vascular endothelial injury through multiple mechanisms. It can reverse endothelial damage caused by excessive NO by inhibiting nitrosative stress and protecting ECs. Additionally, it inhibits the promoting effect of ATP on NO production in vascular ECs and reduces intracellular calcium concentration and eNOS activity, ultimately reducing vascular endothelial injury and stabilizing intravascular homeostasis [58]. In a new animal study utilizing aneurysm and dissection mouse models, quercetin was found to suppress the expression of VCAM-1 and pro-matrix metalloproteinase-9 activity in the aorta of mice, along with reducing macrophage infiltration into the aortic wall. Quercetin also significantly inhibited the enlargement of the abdominal aortic diameter, reduced the incidence of aortic aneurysms, and prevented death from rupture in mice. Moreover, quercetin suppressed the expression of VCAM-1 in response to TNF-α stimulation in human umbilical vein endothelial cells. These findings suggest that quercetin effectively prevents the onset of atherosclerosis-related acute aortic syndromes through its anti-inflammatory properties [74].

Numerous in vivo and in vitro studies have demonstrated that quercetin increases the expression of SIRT1 [75–79]. A study showed that quercetin inhibited endoplasmic reticulum stress through activating the SIRT1/AMPK signaling pathway [80]. In another study, the administration of 20 mg/kg/day of quercetin for 8 weeks effectively reduced lipid deposition in arterial lumina and atherosclerotic lesions, concurrently decreasing the levels of serum ICAM-1, IL-6, and VCAM-1 in the aorta. Moreover, it increased the density of SIRT1 in the aorta of ApoE^−/−^ mice. In in vitro studies, quercetin reduced the expression of senescence-associated β-galactosidase and improved the cell morphology of human aortic endothelial cells (HAECs). Furthermore, quercetin reduced cellular apoptosis, increased mitochondrial membrane potential (ΔΨm) in a dose-dependent manner, and decreased ROS generation [22]. In diabetic rats fed high-fat diet, treatment with quercetin was reported to improve the lipid profile, reduce atherosclerotic lesions, lower the atherogenic index, decrease malondialdehyde (MDA) levels, and increase the activity of enzymatic antioxidants in the carotid artery. Additionally, quercetin suppressed the inflammatory response by reducing NF‑κB and IL‑1β levels, while increasing IL‑10 levels through the AMPK/SIRT1/NF‑κB signaling pathway [21]. In a recent study, quercetin was shown to regulate mitophagy and endoplasmic reticulum stress through the SIRT1/TMBIM6 pathway, inhibiting oxidative stress damage in human cardiomyocytes. The study also revealed that the number of cell apoptosis in the quercetin-treated group was significantly reduced, with increased expression of SIRT1, PGC-1α, and Bcl-2 proteins [81].

### Berberine

Berberine (BBR) is an isoquinoline quaternary alkaloid (or a 5,6-dihydrodibenzo(a,g)quinolizinium derivative) widely used in traditional Chinese herbal medicine isolated from several plants such *Berberis vulgaris* (barberry), *Hydrastis canadensis* (goldenseal), *Coptis chinensis* (Chinese goldthread), *Cortex phellodendri* (Huangbai), and *Rhizoma coptidis* (Huanglian) [82]. Berberine is a yellow powder, odorless with characteristic alkaloid bitterness. It is sparingly soluble in water and slightly soluble in ethanol or methanol; however, the salt form is relatively water-soluble. Berberine can be easily obtained from medicinal plants or through total synthesis. Chlorides or sulfates of berberine are commonly used for clinical purposes [83]. Over the past few decades, berberine has gained significance in traditional Chinese medicine due to its wide range of applications. However, despite its strong pharmacological effects, its oral bioavailability is exceptionally low [84].

In recent years, numerous in vivo and in vitro studies have demonstrated that berberine effectively reduces plasma levels of TC, TG, LDL-C, and non-HDL-C while elevating HDL-C. It also mitigates lipid and cholesterol accumulation in macrophages [85]. Furthermore, many studies have observed a reduction in atherosclerosis lesions, accompanied by decreased TNF-α, IL-1β, and IL-6 levels, along with increased IL-10 and adiponectin levels [86–89]. Recently, the effects of berberine on trimethylamine *N*-oxide (TMAO) production in the gut microbiota and its impact on plaque development in atherosclerosis were investigated. This research encompassed studies involving animal intestinal bacterial cultures, HFD-fed hamsters, and atherosclerotic patients [90]. Twenty-one patients with atherosclerosis showed an average decrease in plaque score by 3.2% after taking 0.5 g of oral berberine for 4 months. Furthermore, trimethylamine (TMA) and TMAO levels in patients decreased by 38% and 29% in feces and by 37% and 35% in plasma after 4 months of berberine treatment [90]. Another study demonstrated that in C57BL/6 J and ApoE KO mice on a choline-supplemented chow diet, berberine attenuated TMA/TMAO production. This treatment also reduced atherosclerotic lesion areas in ApoE KO mice. Furthermore, berberine exhibited a significant inhibitory effect on TMA formation in the gut microbiota isolated from human fecal samples [91]. The berberine-induced inhibition of TMA/TMAO production was observed in both in vivo and in vitro human-based studies. This has offered novel insights into the mechanisms responsible for the anti-atherosclerosis effects of berberine [90, 91]. In another study, berberine was found to reduce serum lipid levels, counteract hepatic lipid accumulation, improve intima-media thickening, reduce aortic ROS generation, and decrease serum levels of MDA, ox-LDL, and IL-6 in ApoE^−/−^ mice fed a western-type diet for 12 weeks. Additionally, berberine ameliorated endothelial dysfunction and provided protection against atherosclerosis through its involvement in pathways associated with mitochondrial dysfunction, fatty acid β-oxidation, and FXR/RXR activation [92].

Recent studies, both in vivo and in vitro, have focused on the activities of berberine and SIRT1 [24, 93–95] and their underlying anti-atherogenic mechanisms. Berberine promoted autophagy of peritoneal macrophages by activating SIRT1 via the NAD^+^ synthesis pathway, thus promoting transcription factor EB (TFEB) nuclear translocation and deacetylation [94]. Berberine, through the activation of SIRT1 via the NAD^+^ synthesis pathway, promotes the autophagy of peritoneal macrophages. This, in turn, facilitates TFEB nuclear translocation and deacetylation, contributing to its underlying anti-atherogenic mechanisms [23]. Klotho (KL) is an anti-aging protein known to promote health and extend the lifespan of individuals. Deficiency of KL has been correlated with cardiovascular disease, and low expression of KL is considered an early predictor of atherosclerosis [96]. Berberine increased KL expression and significantly reversed the downregulation of SIRT1 in the aging heart. This effect markedly suppressed the development of doxorubicin (DOX)-induced cardiac senescence and protected the aging heart of male Sprague Dawley rats [23]. Furthermore, in H9c2 cells, berberine and KL were found to increase the expression of SIRT1 [23]. Berberine also demonstrated its ability to protect against DOX-induced cardiotoxicity and oxidative stress through the downregulation of SIRT1-mediated p66Shc signaling. This protection was associated with the modulation of ROS both in vivo and in vitro [24].

### Fisetin

Fisetin (3,3′,4′,7-tetrahydroxyflavon) is a naturally occurring compound with the molecular formula C_15_H_10_O_6_. It can be found in various fruits such as apples, strawberries, kiwis, mangoes, grapes, persimmons, and peaches, as well as in vegetables including onions, tomatoes, cucumbers, and kale. Additionally, fisetin is present in nuts and wine [20]. The average daily intake of fisetin is estimated to be 0.4 mg [97]. Fisetin is appreciated for its health-promoting properties and its potential use as a nutraceutical, as demonstrated in pre-clinical studies [98–102]. Fisetin is known for its anti-inflammatory, antioxidant, anti-carcinogenic, anti-allergic, neuroprotective, and cardiovascular preventive properties [103–108].

Fisetin administered as an aqueous solution at a dose of 12.5 mg/kg was shown to reduce atherosclerosis in ApoE^−/−^ mice after 12 weeks. In the aortic sinus, atherosclerotic changes and lipid accumulation were significantly reduced compared to the control group when fisetin was administered. Fisetin also demonstrated the ability to reduce the expression of PCSK9 and LOX-1, as well as aging markers including p21 (cyclin‑dependent kinase inhibitor 1A), p53 (tumor suppressor protein p53), and p16 (multiple tumor suppressor‑1). These transcription factors are associated with apoptosis, cell cycle regulation, and senescence in ApoE^−/−^ mice [25, 109]. Fisetin exhibits anti-inflammatory properties. In a study using macrophages where inflammatory responses were induced with lipopolysaccharide (LPS), fisetin reduced the expression of pro-inflammatory MCP-1, IL-1β, and iNOS. Additionally, fisetin prevented foam cell formation by impacting macrophage recruitment and infiltration through the reduction in the expression or activity of uPA, uPAR, MMP-2, and MMP-9, which are factors associated with macrophage recruitment and infiltration [32].

The relationship between fisetin and SIRT1 has been a topic of discussion for several years [110–112]. In a recent study involving mice, fisetin was found to suppress the activation of Toll-like receptor 4 (TLR4), myeloid differentiation factor 88 (MyD88), and NF-κB and subsequently inactivate pro-inflammatory factors, including IL-6 and TNF-α, with an increased expression of AMPK/SIRT1. Additionally, the study demonstrated that lead (Pb) exposure inhibited the expression of p-AMPK and SIRT1 [26]. Studies demonstrate a link between non-alcoholic fatty liver disease (NAFLD) and atherosclerosis disease [113, 114]. Fisetin regulated lipid metabolism in vitro in FL83B hepatocytes and in male C57BL/6 mice with induced non-alcoholic fatty liver disease. The reduction of serum free fatty acid concentration and decreased lipid accumulation were observed. The mechanism of action indicated a significant increase in the phosphorylation of AMPKα, as well as increased SIRT1 production in liver tissue [112].

### Curcumin

Curcumin, with the chemical formula C_21_H_20_O_6_, is a natural yellow pigment that can be isolated from turmeric (*Curcuma longa* L.). It is also known as diferuloyl methane. The IUPAC (International Union of Pure and Applied Chemistry) name of curcumin is (1E,6E)-1,7-bis (4-hydroxy-3-methoxyphenyl)-1,6-heptadiene-3,5-dione [115]. Researchers became particularly interested in curcumin due to its discovery of anticancer properties by Singh and Aggarwal [116]. Curcumin has demonstrated antioxidant, anti-inflammatory, antiapoptotic, antihypertensive, anti-diabetes, anti-obesity, anti-carcinogenic, and anti-aging properties both in vivo and in vitro [117–119]. Turmeric is a distinctive and essential spice for Indian cuisine. It is estimated that the usual intake of curcumin in India averages 100 mg/day, and studies show that even consumption of up to 8 g/day is safe [120].

Recent in vivo and in vitro studies provide support for the potential of curcumin to reduce atherosclerosis and the pathogenic factors involved in its development [117, 118, 121–123]. A study involving male New Zealand white rabbits demonstrated that the administration of curcumin-phosphatidylserine (100 mg/kg) solid dispersion significantly reduced the intima-media thickness ratio and the grading of atherosclerotic plaque. Rabbits exposed to the 100 mg/kg dosage of curcumin-phosphatidylserine exhibited significantly fewer inflammatory cells in the atherosclerotic lesions compared to the control group [124]. In ApoE^−/−^ mice, curcumin reduced serum levels of LDL-C, TC, and TG and significantly decreased the formation of atherosclerotic plaque in the aorta. It also reduced lipid deposition in the liver and mitigated inflammatory damage in the heart, lung, and kidney [125]. Curcumin has also been shown to play a role in lipid metabolism, inflammation, and autophagy [126–133].

ox-LDL is responsible for impairment of autophagy [134] and the adverse effects of ox-LDL on macrophages can be reversed by the properties of curcumin [122]. Curcumin has demonstrated the ability to restore foam cell autophagy, thereby contributing to the inhibition of atherosclerosis. An in vitro study identified a novel axis, TFEB-P300-BRD4, that appears to be responsible for curcumin’s capacity to inhibit inflammation, reduce lipid content, and regulate autophagy [122]. The anti-inflammatory properties of curcumin have been confirmed on human ECs and monocytes. Curcumin reduced IL-1β in human umbilical vein endothelial cells (HUVECs) and reduced IL-6 and TNF-α in THP-1 cells, resulting in reduced inflammation [33]. Curcumin attenuated VSMC migration by inhibiting NF-κB-mediated NLRP3 expression. It also inhibited NLRP3 expression and reduced IL-1β concentration in VSMCs [135].

The relationship between curcumin and SIRT1 has been a subject of discussion for years. In a study involving a high-fat diet, curcumin inhibited age-related vascular changes by increasing SIRT1 expression and also led to decreased glucose and TC levels [27]. A recent study demonstrated that curcumin treatment induced the activation of the SIRT1/NRF2 pathway and inhibited TLR4 expression in newborn rats. This led to an improvement in the inflammatory condition of necrotizing enterocolitis, with reduced expression of inflammatory factors in the intestinal tissue of NEC newborn rats. Furthermore, curcumin inhibited the expression of inflammatory factors in intestinal epithelial cells induced by LPS/ATP and attenuated the LPS/ATP-induced focal death pathway in intestinal epithelial cells through the SIRT1 pathway [136]. Another study demonstrated that tetrahydrocurcumin, a natural curcumin metabolite, increased the expression of SIRT1 and deacetylated SOD2, both in in vitro and in vivo settings. This effect protected cardiomyocytes against oxidative damage [137].

### Catechins

Catechins (flavan-3-ols) are polyphenols that naturally occur in some vegetables (e.g., legumes) and fruits (lychees, apples, grapes) and in other plant foods, such as teas (green tea (*Camellia sinensis*) and pu-erh), cocoa beans, and buckwheat [138, 139]. Green tea contains the following forms of catechins: ( −)-epigallocatechin-3-gallate (EGCG), ( −)-epicatechin-3-gallate (ECG), ( −)-epigallocatechin (EGC), and epicatechin (EC) [140]. Today, catechins have garnered significant attention from researchers due to their anti-inflammatory, antihypertensive, antibacterial, antioxidant, anti-atherosclerotic, and anticancer properties [139].

Catechins are known to influence vasodilation, a key factor in maintaining proper endothelial function and preventing atherosclerosis development. Studies conducted in HUVECs, bovine coronary artery endothelial cells (BCAECs), and male Wistar rats have demonstrated that catechins can enhance eNOS expression and NO production, offering protection against endothelial dysfunction and vasoconstriction [30, 34]. Catechins also exhibit anti-atherosclerotic properties by influencing cellular aging and apoptosis [34, 141]. In the in vitro part of the study, epicatechin reduced β-galactosidase activity, a marker of cell aging [34]. Researchers found that ( +)-catechin had broad atheroprotective effects, including reducing oxidative stress and inhibiting monocyte and smooth muscle cell migration. It also mitigated inflammation and normalized the lipid profile. In cell studies, it decreased ROS production in THP-1 cells, HUVECs, and HASMCs. In a 3-week study with C57BL/6 J mice on a high-fat diet, ( +)-catechin reduced triacylglycerols, IL-1β, and IL-2 in plasma. It also influenced liver gene expression, particularly genes related to cell proliferation, migration, and lipoprotein levels. The intake of catechins reduced atherosclerotic lesion size and increased plaque stability by 58.87% in LDLR^−/−^ mice [142]. ECG exhibits anti-atherosclerotic effects similar to ( +)-catechin. ECG reduces oxidative stress by lowering MDA levels and increasing SOD activity in both in vitro and in vivo studies. In ApoE^−/−^ mice, ECG reduces lipid accumulation in the aorta and aortic roots, stabilizes atherosclerotic plaques, and decreases MMP-2 and ICAM-1 expression [143]. In addition, ECG’s mechanism of action involves inhibiting the pro-inflammatory NF-κB pathway, particularly the p65 subunit, resulting in the downregulation of inflammatory mediators. ECG also exerts anti-inflammatory and antioxidant effects by interacting with Nrf2 and increasing HO-1 expression [143]. The induction of autophagy and cholesterol efflux by oligomeric proanthocyanidins and ECG through the class III PI3K/beclin1 pathway in foam cells represents a promising therapeutic strategy for combating atherosclerosis. Impaired autophagy is a significant contributor to atherosclerotic disease, and these compounds could potentially address this issue [144].

Many in vitro and in vivo studies have demonstrated that curcumin increased the expression of SIRT1 [95, 145, 146]. A recent study showed that EGCG reduced serum TG, TC, LDL-C, and free fatty acid levels; reduced lipid droplets in hepatocytes; and increased serum HDL-C levels, T-AOC, and SOD activity in hyperlipidemic rats. Additionally, it was shown that EGCG activated SIRT1, activated FoxO1 protein, regulated SREBP-2 protein, and inhibited hepatic cholesterol synthesis with decreased SREBP-2 expression. Also, it was shown that EGCG reduced MDA and increased T-AOC and SOD in the liver, indicating that it improved the body’s antioxidant capacity, reducing the generation of peroxides [147].

## Conclusions

In vitro and in vivo studies, as well as clinical trials in humans, have shown that SIRT1 activation might reduce or prevent atherosclerosis through various mechanisms. SIRT-activating compounds derived from natural sources emphasize the importance of dietary interventions to prevent atherosclerosis. However, it remains unclear whether the effects of these compounds are mostly related to SIRT activation. It is important to determine the correct dose or concentration, as many of the effects are dose-dependent. Since most of the natural compounds described here exhibit pleiotropic effects, establishing a direct link between SIRT1 activation and the prevention or reduction of atherosclerosis is quite challenging. It is also evident that a more robust correlation between health effects and the administration of bioactive compounds needs to be established to understand their biological impact and their direct association with SIRT1 activation.

Additionally, the bioavailability and solubility of these natural compounds is very low. Treatment with higher concentrations of bioavailable, bioactive compounds may result in increased SIRT1-activating action, further substantiating the link between SIRT1, compounds, and their therapeutic effects. Despite the generally encouraging data from in vitro and in vivo studies, supporting molecular evidence that provides clues to these unanswered questions is still lacking. A better understanding of the molecular mechanisms of these natural molecules or their derivatives is needed for their preclinical and clinical usage. Berberine, fisetin, curcumin, catechins, and resveratrol have shown the ability to activate sirtuins, particularly SIRT1. Our review focused on recent studies investigating the atheroprotective effect and the underlying molecular mechanisms. The conclusion of many studies is that it is necessary to better understand the molecular and epigenetic mechanisms of these compounds to prevent or treat atherosclerosis in humans.

## Abbreviations

*4-HNE*: 4-Hydroxynonenal
*AMPK*: AMP‐activated protein kinase
*ANP*: Atrial natriuretic peptide
*ApoE*^−^^/^^−^: Apolipoprotein E knockout
*Bcl-2*: B-cell lymphoma 2
*CCL2*: Chemokine 2
*CXCL1*: Chemokine 1
*FoxO1*: Forkhead box protein O1
*FXR*: Farnesoid X receptor
*ICAM-1*: Intercellular adhesion molecule 1
*IκBα*: Nuclear factor of kappa light polypeptide gene enhancer in B-cell inhibitor alpha
*IL*: Interleukin
*KC*: Keratinocyte chemoattractant
*LXRα*: Liver X receptor alpha
*MCP-1*: Monocyte chemoattractant protein-1
*MHC*: Myosin heavy chain
*Nrf2*: Nuclear factor erythroid 2–related factor 2
*p-AMPK*: Phosphorylated AMP‐activated protein kinase
*PCSK9*: Proprotein convertase subtilisin/kexin type 9
*PI3K-Akt*: Phosphatidylinositol 3‑kinase/protein kinase B
*RXR*: Retinoid X receptor
*SOD*: Superoxide dismutase
*SR-BI*: Scavenger receptor class B type I
*T-AOC*: Total antioxidant capacity
*TIMP-1*: Tissue inhibitor matrix metalloproteinase 1
*TMA*: Trimethylamine
*TNF-α*: Tumor necrosis factor α
*Trib1*: Tribbles homolog 1
*VCAM-1*: Vascular cell adhesion protein 1
